# Exploiting temporal continuity of views to learn visual object invariance

**DOI:** 10.3389/fnins.2013.00026

**Published:** 2013-03-01

**Authors:** Evgeniy Bart, Jay Hegdé

**Affiliations:** ^1^Palo Alto Research CenterPalo Alto, CA, USA; ^2^James and Jean Culver Vision Discovery Institute and Department of Ophthalmology, Medical College of Georgia, Georgia Regents UniversityAugusta, GA, USA

In an ever-changing visual world, the appearance of visual objects changes constantly. Yet, our perception of a given object stays robust despite the variations in the image. The mechanisms that implement this perceptual invariance are partially known (e.g., Logothetis et al., 1995). It is also known that these mechanisms are at least in part learned from experience, but the learning processes involved are not yet fully understood.

Theoretical studies have suggested that the visual system may achieve this learning by using temporal association. The underlying idea is that the object currently in view is likely to be the same object that was in view a moment ago, even if its appearance has changed in the meantime due to factors such as relative motion. The visual system may therefore learn an association between potentially different-looking images if they appear in temporal succession. Learning invariance in this manner is known as temporal trace learning (Földiák, 1991; see Rolls, 2012, for a review), and it is the focus of the study by Isik and colleagues.

Previous psychophysical studies by other groups have shown that the visual system can indeed exploit temporal continuity to learn invariance (Wallis and Bulthoff, 1999, 2001; Cox et al., 2005). Studies have also shown that, as predicted by the trace learning rule, the visual system can be made to learn false invariance by simulating false temporal continuity between distinct objects. Under the right experimental conditions, adult subjects can be made to confuse two completely different objects with each other after as little as 1 h of training (Li and Dicarlo, 2010). This suggests that the visual system can and does rely on temporal continuity of objects to infer invariance, and that the ability to learn using this method persists in adulthood.

It also raises, however, a troubling question. If the visual system can be made to learn false invariance in this way, what is to prevent false invariances from disrupting object recognition all the time? This is a real possibility, because spurious temporal continuities are not at all uncommon under natural viewing conditions. Rapid movement (of the object or the observer) or sudden occlusions may cause distinct objects to be observed in close temporal proximity. Note that although some other learning rules (e.g., continuous spatial transformation learning; Ullman, 1996) may be more robust to this type of disruptions, it is known that at least in some cases basic temporal association learning is used by the visual system (Li and Dicarlo, 2010). So what minimizes this invariance disruption and keeps object recognition robust and stable?

The study by Isik and colleagues provides a compelling potential answer. The authors describe a plausible network model of the primate visual cortex in which simulated visual cortical neurons learn invariance by using a version of the temporal trace rule. The model is based on the previously described HMAX model (Serre et al., 2007). HMAX is a hierarchical feed-forward model which consists of multiple layers of visual neurons. Each layer extracts increasingly complex shape features of the image based on the input from the lower layer, and passes it on to the next higher layer [for details, see Figure 1 of Serre et al. (2007)]. Thus, each neuron in a given layer “listens to,” and integrates information from, multiple neurons in the previous layer, so that neurons in the topmost layer, arguably corresponding to those in the primate inferotemporal cortex, collectively contain a complex representation of the objects in the various input images.

The authors augmented HMAX to incorporate a simplified, but effective, implementation of learning by temporal association, called the “modified trace rule.” This augmented model was able to reproduce a diagnostic feature of invariance learning: when trained with smooth temporal variations of a given object, such as a face [see Figure 1, *top left*, of Isik et al. (2012)], the neurons in the topmost layer of the network, individually and collectively, did learn an invariant representation of that object.

The authors then studied the behavior of the model when trained with image sequences that contained false temporal continuity. In each such sequence, images at all positions showed the same object (e.g., a face), except for one position (called the “swap position”) that showed a different object [e.g., a car; see Figure 1, *top right*, of Isik et al. (2012)]. As expected, invariance tuning of each cell trained with such a sequence was disrupted, with the cell responding to the main object at most locations, but responding more strongly to the swap object at the swap location. Thus, individual cells did faithfully learn false invariance.

However, the neuronal population as a whole still robustly represented all stimuli. The reason is that in the simulated experiment (as under natural viewing conditions), the disruptions were relatively infrequent, and their locations were random. As a result, for any given location, the majority of cells responded consistently, thus producing consistent population-level encoding. The authors found that disruptions of continuity in the training sequences did not appreciably affect the overall population response until the amount of altered exposure was as high as 25%. As expected, robustness of invariance improved as the size of the neural population increases. This confirms the intuition that invariances that rely on larger neural populations are harder to disrupt. Altogether, the central contribution of this model is demonstrating that a highly plausible implementation of trace learning can capture known key characteristics of object invariance, including the conditions in which it remains robust and the conditions in which it does not.

The computational framework the authors have developed can also be used to address additional important questions about invariance. For example, it can be used to test whether invariance can be disrupted more easily when recognizing more similar objects (e.g., when distinguishing between several faces, as opposed to between cups and sailboats). It can also be used to compare invariances to various kinds of transformations, such as out-of-plane rotations or illumination changes.
